# Untargeted Blood Lipidomics Analysis in Critically Ill Pediatric Patients with Ventilator-Associated Pneumonia: A Pilot Study

**DOI:** 10.3390/metabo14090466

**Published:** 2024-08-23

**Authors:** Christina Virgiliou, Olga Begou, Argyro Ftergioti, Maria Simitsopoulou, Maria Sdougka, Emmanuel Roilides, Georgios Theodoridis, Helen Gika, Elias Iosifidis

**Affiliations:** 1 Analytical Chemistry Laboratory, Department of Chemical Engineering, Aristotle University of Thessaloniki, 54124 Thessaloniki, Greece; 2 Biomic Auth, Bioanalysis and Omics Lab, Centre for Interdisciplinary Research of Aristotle University of Thessaloniki, Innovation Area of Thessaloniki, 57001 Thermi, Greece; mpegolga@chem.auth.gr (O.B.); gtheodor@chem.auth.gr (G.T.); gkikae@auth.gr (H.G.); 3 Department of Chemistry, Aristotle University of Thessaloniki, 54124 Thessaloniki, Greece; 4 Infectious Diseases Unit, 3rd Department Pediatrics, School of Medicine, Faculty of Health Sciences, Aristotle University of Thessaloniki, Hippokration General Hospital, 54642 Thessaloniki, Greecesimitsop@auth.gr (M.S.); roilides@auth.gr (E.R.); iosifidish@auth.gr (E.I.); 5 Pediatric Intensive Care Unit, Hippokration General Hospital, 54642 Thessaloniki, Greece; mariasdougka@gmail.com; 6 Laboratory of Forensic Medicine and Toxicology, School of Medicine, Aristotle University of Thessaloniki, 54124 Thessaloniki, Greece

**Keywords:** lipidomics, metabolomics, VAP, HRMS, pediatric patient

## Abstract

This study aims to explore the diagnostic potential of blood lipid profiles in suspected ventilator-associated pneumonia (VAP). Early detection of VAP remains challenging for clinicians due to subjective clinical criteria and the limited effectiveness of current diagnostic tests. Blood samples from 20 patients, with ages between 6 months and 15 years, were collected at days 1, 3, 6, and 12, and an untargeted lipidomics analysis was performed using a Ultra high Pressure Liquid Chromatography hyphenated with High Resolution Mass Spectrometry UPLC-HRMS (TIMS-TOF/MS) platform. Patients were stratified based on modified pediatric clinical pulmonary index score (mCPIS) into high (mCPIS ≥ 6, *n* = 12) and low (mCPIS < 6, *n* = 8) VAP suspicion groups. With the untargeted lipid profiling, we were able to identify 144 lipid species from different lipid groups such as glycerophospholipids, glycerolipids, and sphingolipids, in the blood of children with VAP. Multivariate and univariate statistical analyses revealed a distinct distribution of blood lipid profiles between the studied groups, indicating the potential utility of lipid biomarkers in discriminating VAP presence. Additionally, specific lipids were associated with pharyngeal culture results, notably the presence of Klebsiella pneumoniae and Staphylococcus aureus, underscoring the importance of lipid profiling in identifying the microbial etiology of VAP.

## 1. Introduction

Ventilator-associated pneumonia (VAP) is one of the most common nosocomial infections in critically ill children in pediatric intensive care units (PICUs). VAP is a severe complication, leading to increased morbidity, mortality, and healthcare costs [1]. Timely and accurate diagnosis is essential for early initiation of appropriate antimicrobial treatment [2,3,4]. Clinicians face significant challenges in diagnosing VAP early on, primarily due to the reliance on subjective clinical criteria and the limited accuracy of existing diagnostic tests. While most VAP definitions are based on adult ICU research, pediatric patients require distinct classifications due to their unique physiologies and pathologies [1]. Few studies have focused on the risk factors for pediatric VAP, and there is still no gold standard for a VAP definition and diagnosis in children. Current diagnostics rely on clinical assessment, radiological imaging, and microbiological cultures of respiratory samples. These methods exhibit high inter-variability and only moderate sensitivity and specificity. Microbiological confirmation can take several days, resulting in overtreatment with antibiotics until the specific pathogen is identified [5]. New diagnostic tools—faster, more reliable and robust, and less invasive—are required to differentiate patients with different severities or characteristics of VAP, enabling potential therapeutic targets and personalized treatment strategies.

Omics approaches can offer new advantages in diagnostics as complex pathologies can be more accurately diagnosed via the readout of the whole metabolic phenotype than with the current one-dimensional, single-marker diagnostic procedures. Metabolomics represents a rapidly evolving discipline focused on the holistic analysis and study of small endogenous molecules (molecular weight, MW < 1500 Da), commonly referred to as “metabolites” [6]. Lipidomics, a subfield of metabolomics, involves the study of the lipidome or the total lipid content within a cell, organ, or biological system [7]; however, only a few studies have been conducted in pneumonia and, more particularly, in children cohorts. The majority of the studies have been focused on the analysis of small, volatile organic compounds (VOCs) in the exhaled breath of hospitalized ICU adult patients or in strain liquid cultures using Gas Chromatography coupled to different Mass Spectrometry detectors, GC-MS or GC-qTOFMS analytical platforms [8,9,10,11,12,13,14]. The VOC’s metabolic profile was found to be differentiated between various disease states, including VAP; thus, breath samples are found to be an ideal, non-invasive matrix for VAP diagnosis and/or prognosis. A few studies explored urine and serum metabolic profiles of the same population groups using Reversed Phase Liquid Chromatography RPLC-qTOFMS and Nuclear Magnetic Resonance 1D 1H NMR, respectively [15,16]. Only Laiakis et al. investigated possible alterations in the plasma and urine metabolic profiles of Gambian children with VAP compared to healthy controls using LC-qTOF MS [17].

The objective of this study was to investigate the diagnostic potential of blood lipidomic biomarker signatures for identifying VAP in hospitalized, pediatric, critically ill patients. By examining lipidomic profiles, this study aimed to provide a more reliable and objective method for early VAP diagnosis that could potentially improve patient management and ultimately prognosis.

## 2. Materials and Methods

### 2.1. Study Population

The study design and patient population enrolled in the study are mentioned in detail by Sdougka et al. [18]. Briefly, children aged from 1 month to 16 years with clinical suspicion of VAP and on mechanical ventilation for at least 48 h were eligible for the study, and informed consent was asked by parents/caregivers.More information about study population can be found in Table 1.

Enrollment criteria included different levels of clinical suspicion. Step one was a purulent respiratory or positive bronchial aspirate culture and initiation of antimicrobial agents; an increased oxygen requirement (>20%) with fever, hypothermia, leukocytosis, or leukopenia, and an initiation of antimicrobial agents; and new lung infiltrates on radiology with at least two additional criteria such as fever, hypothermia, increased oxygen requirement, purulent secretions, abnormal white blood cell count, and elevated C-reactive protein CRP. The exclusions criteria included a lack of consent, unlikely survival beyond 48 h, pregnancy in adolescent females, and a body weight < 3 kg. 

On day 1 (step 2), all the patients enrolled were further assessed using the modified CPIS (mCPIS) tool and categorized into high (mCPIS ≥ 6) and low (mCPIS < 6) VAP suspicion groups. Twelve children were allocated to high VAP suspicion and eight to low VAP suspicion, according to the mCPIS. 

Pharyngeal cultures were obtained on days 1, 3, 6, and 12. Pathogen identification was performed using the Vitek^®^ 2 automated system (Biomerieux, Marcyl’Etoile, France). 

### 2.2. Chemicals and Reagents

LC-MS grade acetonitrile (ACN), methanol (MeOH), and 2-propanol (IPA) were procured from Sigma-Aldrich (St. Louis, MO, USA), while methyl-tert-butyl-ether (MTBE; ≥99%), ammonium formate, and formic acid (FA, >99% LC-MS grade) were supplied by Chem-Lab NV (Zedelgem, Belgium). Pure water (H_2_O, 18.2 MΩ cm^−1^) was purified using a Milli-Q device (Millipore Purification System, Merck Darmstadt, Darmstadt, Germany). 

### 2.3. Blood Samples

Blood samples of the children were collected at four specific time points: days 1, 3, 6, and 12 of hospitalization at the ICU following the onset of the event (VAP suspicion). They were then divided into respective aliquots for all analyses performed (hematological, biochemical, metabolomics/lipidomics), and stored at −70 °C until the LC-HRMS analysis.

### 2.4. Sample Preparation

Blood samples were left to thaw at 4 °C prior to the analysis. Fifty microliters of sample were diluted with 700 μL of ice-cold MeOH:MTBE, 1:3 *v*/*v* (−20 °C), vortexed for 5 min, and centrifuged at 25,200× *g* for 20 min at 4 °C. Sixty hundreds microliters of the organic phase were then transferred to an Eppendorf tube and evaporated to dryness under a vacuum (SpeedVac, Eppendorf Austria GmbH, Vienna, Austria). The residue was reconstituted with 150 μL of H_2_O:ACN:IPA (1:1:3, *v*/*v*), and the clear supernatant was transferred into a LC-MS/MS glass vial, placed in an autosampler at 6 °C, and subjected to an RPLC-HRMS analysis. 

Quality control (QC) samples were prepared by mixing equal volumes of all analyzed clinical samples (20 μL). Phenotypic QC samples were also prepared for high and low VAP suspicion groups by mixing equal volumes of the respective samples. All QC samples were treated as mentioned above. From the initial QC samples, diluted QCs (1:2, 1:4 and 1:8, *v*/*v*) were also prepared for the evaluation of the dilution factor of the detected features in both positive and negative ionization mode.

### 2.5. RPLC-TOFMS Analysis

An analysis was performed in a UHPLC Elute chromatography system coupled to a tims (time of flight) TOF mass spectrometer (Bruker, Billerica, MA, USA), operating in positive and negative electrospray ionisation ESI ionization mode. For chromatographic separation of all analytes, an ACQUITY UPLC CSH C18 (2.1 × 100 mm, 1.7 μm) column was used, equipped with an ACQUITY UPLC Van-Guard pre-column of the same packing material, (Waters Ltd., Elstree, UK). The binary mobile phase system consisted of (a) ACN:H_2_O (60:40, *v*/*v*), 10 mM ammonium formate and 0.1% formic acid, and (b) IPA:ACN (90:10, *v*/*v*), 0.1% formic acid. The gradient elution program was set as follows: 60–57% A (0.0–2.0 min), 57–50% A (2.0–2.1 min), 50–46% A (2.1–12.0 min), 46–30% A (12.0–12.1 min), 30–1%A (12.1–18 min), 1–60% A (18.0–18.1 min), and 60% A (18.1–20.0 min). Flow rate and column temperature were 0.3 mL/min and 55 °C, respectively. Before and after each injection, the needle was conditioned with both a weak and strong wash cycle (1500 μL each), consisting of ACN:H_2_O (60/40, *v*/*v*) and IPA:ACN (90:10, *v*/*v*), respectively. Injection volume was 3 μL for both positive and negative ESI mode.

Data-dependent acquisition (DDA) experiments were performed for the analyses of all blood samples. The parameters in ESI included capillary voltage for both modes at ±4.5 kV, and dessolvation parameters were 200 °C, 10 L/min and 2 bar for dry temperature, dry gas, and nebulizer gas, respectively. Dynamic spectra acquisition was applied for auto MS/MS analysis with a frequency of minimum 6 and maximum 10 Hz. Regarding mass fragmentation, collision energy was set at 20 V for *m*/*z* below 100, 30 V for *m*/*z* 100–1000, and 40 V for *m*/*z* 1000–2000 *m*/*z*. Deflection delta and transfer time were set to 80 V and 54 μs, respectively, and collision RF (Radio Frequency) was set to 1100 Vpp. Sodium formate 10 mM was used as a calibrant mixture and was directly infused into the MS at a 30 µL/h and acquired in the first 0.5 min of each injection.

Quality control samples (QCs) were used throughout the analytical batch in order to evaluate the system’s repeatability and analytical performance. QC samples were analyzed seven times at the beginning of the analytical batch for system equilibration and then at every other 10 blood samples. Phenotype QCs and diluted QCs were also analyzed after the system’s equilibration. 

### 2.6. Data Handling and Statistical Analysis

All obtained raw data underwent recalibration based on sodium formate clusters using a vendor’s software “Data Analysis” (version 5.3, Bruker, Bremen, Germany) and then was converted to a .mzML file format using MSConvert v. 3.0.20133 (ProteoWizard). Chromatographic peak detection, retention time alignment, and feature grouping were performed with XCMS (version 3.2.0) in the R programming environment, where extracted feature values were reported as peak areas. Normalization of the raw data was performed using quality control (QC) samples, support vector regression (QC-SVRC), and a radial basis function kernel to correct the intra-batch effect as proposed by Kuligowski et al. [19]. Features further assessed were present in more than 80% of real samples in each group, and their coefficient variation (CV) in the QC sample was less than 0.3%. Zero or missing values were replaced with half of the respective minimum feature value of the respective group, and all raw data were log-transformed. For univariate statistical analysis, the advanced software GraphPad Prism 8.0.1 for Windows (GraphPad Software, La Jolla, CA, USA) and SPSS Statistics for Windows, version 29.0.2.0 (IBM Corp., Armonk, NY, USA) were used. Data were checked for normal distribution based on the Shapiro–Wilk test (significance level, alpha = 0.05), and respective parametric or non-parametric tests (Mann-Whitney or Kruskal-Wallis) were applied, followed by false discovery rate (FDR) correction (Benjamini, Krieger, and Yekutieli). The statistical significance threshold value was defined as *p*-value < 0.05, and variation between lipids was illustrated as a box and whisker plot. The area under the curve–receiver operating characteristic (AUC–ROC) curves and the logarithm base two-fold change (log2FC) were calculated setting the initial/control value to low, or Klebsiella pneumoniae group, and the final value to high, or Staphylococcus aureus group.

For multivariate statistical analysis, unsupervised principal component analysis (PCA) and orthogonal partial least squares-discriminate analysis (OPLS-DA) in UV scaling were performed using SIMCA P+ 13.0 software (Umetrics, Malmö, Sweden). The validity of the constructed models was based on their R2X, R2Y, and Q2Y values, permutation plots, and *p*-value of CV-ANOVA (cross validation, R2X: fraction of the variation in X explained by the model, R2Y: total sum of variation in Y explained by the model, and Q2Y: goodness of prediction). Different visualization plots, as VIP and “S-plot” graphs, were also assessed for the evaluation of possible biomarkers, with lipids meeting the criteria of *p* > |0.05| and *p* (corr) > |0.5| were considered statistically significant.

### 2.7. Lipid Annotation

Lipid species were firstly identified using Lipostar2 (v.2.0.2, Molecular Discovery Ltd., Hertfordshire, UK) [20] with the LIPID MAPS mass spectra library (version September, 2021) [20] and MSDial software using MS-DIAL LipidBlast library (version 68) [21]. Raw or converted .abf files were imported and aligned with the default settings. Peak picking was executed automatically by the Savitzky–Golay algorithm, employing window size of seven, degree of two, one multi-pass iteration, and a minimum signal-to-noise (S/N) ratio of three. The alignment of lipid profiles was performed by configuring the mass accuracy to 10 ppm and the retention time (RT) tolerance to 0.2 min. Other parameters, such as “Retain lipids with isotopic pattern” and “Retain lipids with MS/MS”, were employed to retain only the compounds exhibiting isotopic patterns and MS/MS in order to be used for possible annotation. Only the lipids with a quality rating of 3–4 stars were accepted and used further for statistical analysis. MS Dial parameters were set as follows: mass accuracy of 0.01 Da and 0.05 Da in MS1 and MS2, respectively, for spectral centroiding, and the identification score cutoff was set to 70%. Identified lipids and possible annotations of both software integrated, and their accuracy, was verified by plotting the retention time of specific lipid species against their Kendrick mass defect relative to the hydrogen base using an in-house script written in R. To gain a thorough understanding of the retention time mapping process for different lipid (sub) classes, one can refer to the detailed description provided by Lange et al. [22].

## 3. Results

### 3.1. Lipid Profiling by LC-TOF-MS

**Figure 1:**
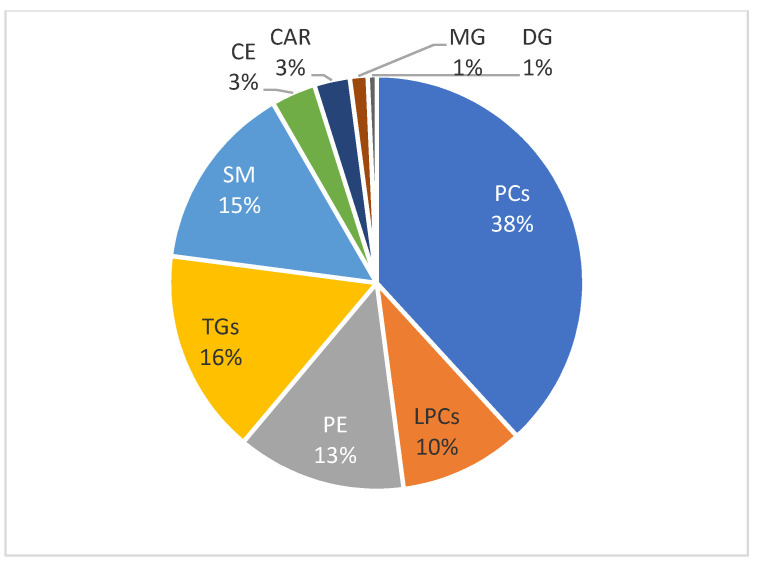
Lipid subclasses quantified in the blood of children with VAP based on lipid analyses. Abbreviations: CAR: Carnitines, SM: Sphingomyelins, LPC: Monoacylglycerophosphocholines, PC: Diacylglycerophosphocholines, PE: Diacylglycerophosphoethanolamines, CE: Cholesterol Esters, DG: Diglycerides, MG Monoglycerides, and TG: Triglycerides.

Raw data processing by XCMS revealed a wealth of features; 2835 ion signals in positive and 1446 in negative ion mode were considered for further analysis after quality control filtering. An untargeted lipidomics workflow facilitated the identification of 144 lipid species, which were then subjected to univariate and multivariate statistical analyses. The identified lipid species in blood plasma comprised 2.7% fatty acyls (4 carnitines), 61.1% glycerophospholipids (88 species), 18% glycerolipids (26 species), 15% sphingolipids (21 species), and 3.4% sterols (5 species). Figure 1 illustrates the subclasses of lipids species quantified in the blood of children with VAP. A detailed table, with the identifications of detected lipids, the chemical formulas, the monoisotopic masses, and the retention time, is presented in Table S1. 

### 3.2. Quality of the Analytical System

**Figure 2:**
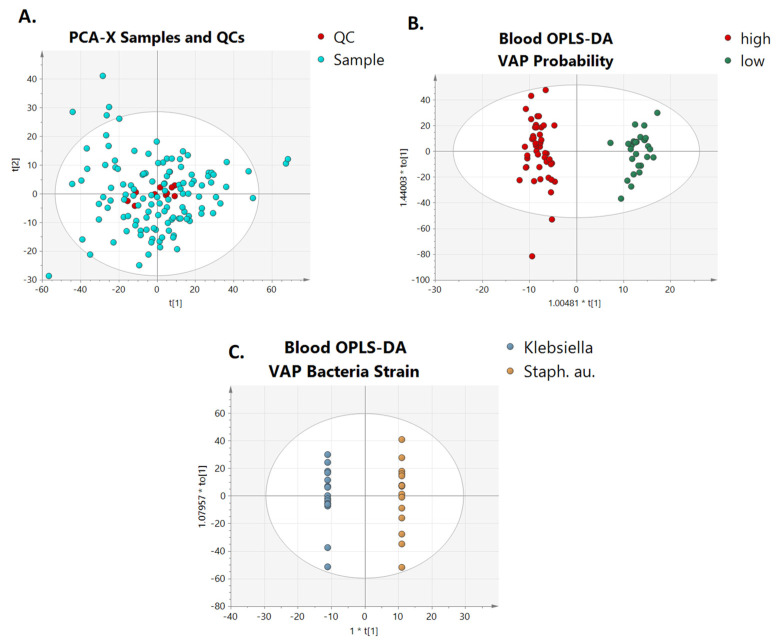
(**A**) PCA-X score plots of all blood samples collected at different time points and QC samples. QC samples are presented with a red color and are clustered together, and OPLS−DA score plot showing the classification of (**B**) high and low VAP suspicion and (**C**) different bacteria strain isolation in pharyngeal cultures based on the blood lipidome.

Initially, to assess the quality, repeatability, and robustness of the analytical system, QC samples were evaluated (Figure 2A) after QC-SVRC normalization, filtering, and exclusion of the ions with % RSD > 30. The score plot demonstrated the clustering of the QC samples, indicating the stability of the analytical system providing a map of how the samples relate to each other. The first component explains 36% of the variation, and the second component explains 44%.

### 3.3. Multivariate Analysis of Lipidomics and VAP Suspicion: High or Low

To investigate any possible lipidomic-based signature associated with the presence of VAP, a multivariate statistical analysis was performed based on the semi-quantified lipid species. The unsupervised PCA scores-plot model constructed could not clearly classify the studied groups. However, separation of the children according to the mCPIS to low and high VAP groups could be achieved by partial least squares-discriminant analysis (PLS-DA). OPLS-DA analysis between the studied groups revealed strong differentiation between children with different CPIS scores for VAP as illustrated in Figure 2B. A combined multivariate and univariate analysis revealed significant perturbations, particularly in response to high and low VAP suspicion groups. All the statistically significant lipids were observed at decreased levels in the blood of children with low VAP mCPIS score. Phosphatidylcholines (LPC 20:5, PC 32:1, PC 32:2, PC 34:1, PC 34:2, PC 34:2, PC 34:3, PC 35:2, PC 36:1, PC 37:3, PC 38:6) sphingomyelins (SM 32:1, SM 34:0, SM 38:1, SM 40:1, SM 40:2, SM 41:1, SM 42:2), and glycerolipids (DG 36:3, TG 46:1, TG 48:1, TG 48:2, TG 48:3, TG 50:4, TG 50:3) were among the main lipid species with their abundances significantly different between the studied groups. 

### 3.4. Multivariate Analysis for Lipidomics and Isolation of Targeted Respiratory Bacterial Pathogens

In addition, we assessed correlations between the blood lipid content of children with VAP suspicion and the bacterial species isolated in pharyngeal swab cultures. The pharyngeal cultures of children showed the presence of pathogens: *K. pneumoniae* in four children, *S. aureus* in four children, and *Acinetobacter* spp., *Stenotrophomonas maltophilia*, and *Candida* spp. in one child each. The rest of the cohort had a negative culture, suggesting that VAP suspicion was clinically, rather than microbiologically, driven. Multivariate statistical analysis was also performed to assess if the bacterial species correlated with the lipid profile in children with VAP suspicion. The PCA scores plot model did not reveal any clear classification of the samples according to the detected bacteria; however, OPLS-DA (Figure 2C) displayed distinct clustering of the studied groups, indicating the correlation of the lipid profile with the presence of different bacterial strains. Table 2 presents the cross-validation parameters of the aforementioned scores plot models. Based on univariate and multivariate analyses, nine lipids were identified to contribute to this classification. Three diglyceride species, namely, DG 16:1_18:0, DG 18:0_18:1, DG 18:1_18:1, eight phosphatidylcholines PC 33:2, PC 34:3, PC 36:4, PC 36:4, PC 37:3, PC 38:4, PC O-34:0 and PC O-38:4, four sphingomyelins SM 34:1, SM 36:2, SM 38:2, SM 40:1, five triacylglycerols TG 48:0, TG 50:1, TG 52:5, TG 54:5, TG 54:6, and the cholesterol ester CE 20:4, demonstrated a significant impact in the discrimination of the two groups, as all of them, with the exception of SM 34:1, were found to be elevated in the blood plasma of children with cultures positive for *S. aureus*. 

**Figure 3:**
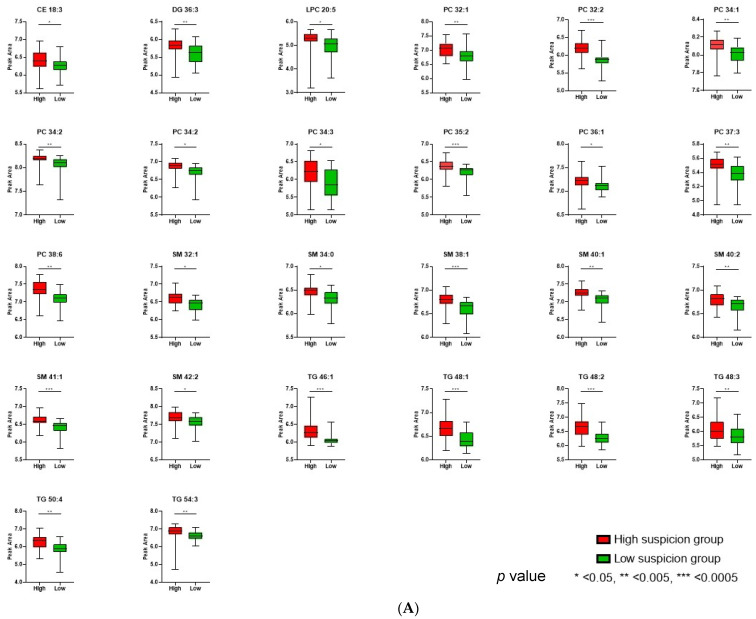

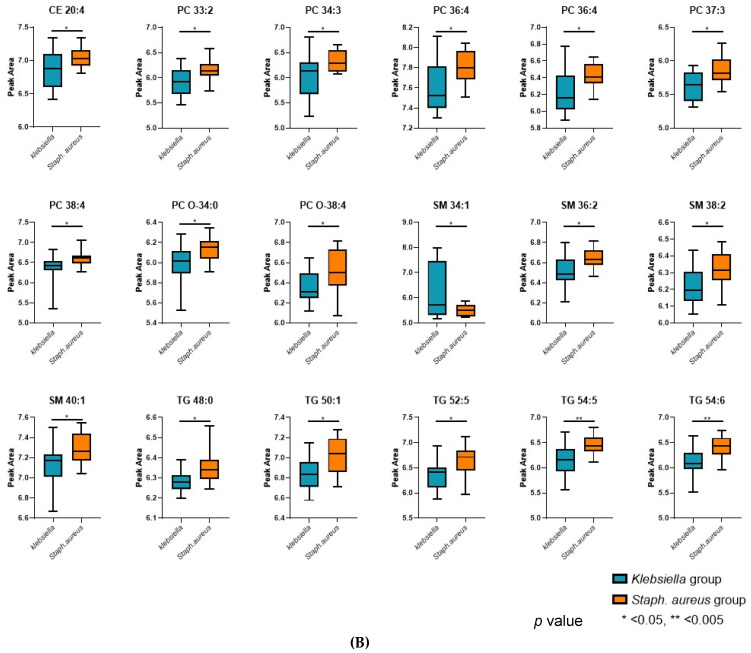
(**A**) Box plots of all significantly altered plasma lipids in children with high (red) and low (green) suspicion in VAP. Data are plotted as median with range. (**B**) Box plots of all significantly altered plasma lipids in children with Klebsiella (blue) and *S. aureus* (orange) bacteria according to pharyngeal culture results. Data are plotted as median with range. (phoscholines PCs, sphyngomyelins SMs, triacylgleycerolsin TGs, Diacylglycerols (DGs, and cholester ester CE).

Statistically differentiated lipid species, derived from all comparisons, are provided in Table 3 and Table 4, along with the respective *p*-values < 0.05, VIP scores, Log2FC, and their lower and upper bounds of the 95% confidence intervals (CI). Figure 3A,B illustrate box plots of all altered metabolites found in the respective comparisons. 

## 4. Discussion

Lipids were found to participate in a variety of processes intimately connected with the pathogenesis of pneumonia and related infectious diseases. The lungs are essential respiratory organs, and lipids play crucial roles in the development and progression of lung diseases. Inflammation and infections induce a plethora of alterations in lipid metabolism that could initially reduce inflammation or fight infection. To the best of our knowledge, this is the first time that the contribution of blood lipids in VAP diagnosis is assessed in critically ill patients. Only a few studies have assessed the relationship between lipid metabolism and pneumonia, and in most of the cases, these studies have focused on community-acquired pneumonia. In these cases, distinct lipid profiles consistently appeared in numerous patients, accompanied by varying airway inflammatory statuses and disease severities. Similarly, in our study, there were differences in lipid metabolism in patients with high or low VAP probability. 

In this study, we characterized the blood lipidome of 20 children with VAP suspicion, including those with high- and low-grades of suspicion according to predefined clinical, laboratory, and imaging criteria (mCPIS score). In addition, given the significant number of children whom tested positive for Klebsiella and Staphylococcus aureus, these groups were further analyzed to identify potential lipid markers associated with these bacteria. Blood untargeted lipidomic analysis revealed extensive variations in phospholipids, sphingolipids, and glycerolipids levels in all these patient groups. In particular, this study identified certain phospholipids, sphingolipids, and glycerolipids at significantly increased abundances in children with low or high VAP suspicion according to mCPIS classification score. 

Cholesterol, sphingomyelins (SM), and phosphatidylcholines (PC) contribute to the creation of the immunological synapse. Additionally, lipids are crucial in activating macrophages, supporting NK (natural killer) cell functionality, and guiding the differentiation and activity of T and B effector cells. Lipid-based signaling is known to regulate various processes linked to inflammation and damage related to infections, such as apoptosis, autophagy, and pulmonary fibrosis [23]. 

Sphingolipids are essential components found in the mucous secreted by the alveolar epithelium, which helps protect lung tissue from invading pathogens [24]. Studies have indicated that Mycoplasma pneumoniae infection in the lungs can trigger the production of autoantibodies against glycosphingolipids, suggesting a potential role of sphingolipids in promoting lung inflammation [25,26]. This raises the possibility that sphingolipids could contribute to pulmonary inflammation during infection. Sphingomyelins are essential cellular components that participate in numerous processes such as cell division, cell proliferation, and autophagy. They also help balance pro- and anti-inflammatory lipids and regulate immune responses in lung tissues [27,28]. Inflammatory lung diseases like pneumonia and chronic obstructive pulmonary disease involve altered sphingomyelin metabolism. This alteration triggers the activation of inflammatory cells and the release of mediators, worsening tissue damage and inflammation [29]. Moreover, abnormalities in sphingomyelin metabolism are associated with pulmonary fibrosis, a serious lung condition characterized by an excessive growth of connective tissue that impairs respiratory function. In pulmonary fibrosis tissues, levels of sphingomyelin are heightened, potentially promoting fibroblast proliferation and collagen synthesis, contributing to the progression of pulmonary fibrosis [30]. In our study, all the SM species, with the exception of SM 42:2, presented AUC values > 0.70, indicating that SMs might be a good indicator for the discrimination of children according to VAP severity. 

**Figure 4:**
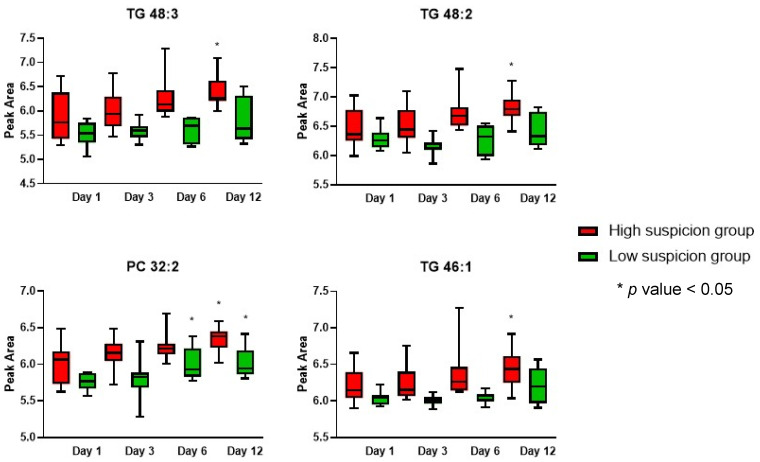
Box plots of significantly altered PCs (phosphocholines) and TGs (triacylglycerols) lipids that presented an AUC value > 0.8 at the two studied groups at the different time points. Asterisks indicate a *p*-value < 0.05 compared to time point 1.

In order to identify significant lipid markers that could aid in VAP diagnosis, the entire set of blood samples was utilized for comprehensive multivariate and univariate statistical analyses to evaluate potential biomarkers. Further longitudinal comparisons among the different time points, along with dynamic monitoring of the lipid abundance, were conducted to provide additional insights and enhance the predictive accuracy for disease severity. Lipid markers with an AUC greater than 0.8 were subjected to further evaluation to identify any trends related to the timing of the sample collection (Figure 4). We found that all triglycerides (TGs) displayed a statistically significant upward trend over time (comparing day 1 to day 12) in the high VAP suspicion group (*p*-value < 0.05). This trend was not observed in the low suspicion group, indicating differences between these two groups. This finding could suggest that monitoring TG levels over time could be a valuable tool in assessing VAP diagnosis in pediatric patients. Such dynamic changes in lipid abundance could potentially serve as robust biomarkers for VAP suspicion and diagnosis, ultimately contributing to better clinical outcomes.

The relationship between phospholipid metabolism and inflammatory diseases has attracted increasing attention in recent years. Our study found that blood phosphocholines and lyso-phosphocholine concentrations were significantly increased in children with high VAP suspicion and significantly decreased in children with positive *K. pneumoniae* cultures. Fluctuations in the abundance of these lipids lead to the change of glycerophospholipid metabolism, which is closely related to the occurrence and development of many diseases including inflammation [31]. The results of the present study suggest that blood phospholipids not only have a strong VAP biomarker potential with AUC ranging from 0.69 for PC 36:1 and PC 32:1 to 0.81 for PC 32:2, but fluctuations in its levels may also be closely related to bacterial infections. Different pathogens may induce different phospholipid metabolism alterations [32]. 

Inflammation and infections can cause shifts in lipid metabolism. While initially beneficial for combating infection or reducing inflammation, prolonged changes may increase the risk of developing atherosclerosis. Common effects include decreased levels of HDL (high-density lipoprotein) cholesterol and increased triglycerides. Inflammation leads to an increase in lipoprotein (a) levels due to an enhanced apolipoprotein (a) synthesis. LDL levels often decrease, but there is an increase in low density LDL particles due to the exchange of triglycerides from triglyceride-rich lipoproteins to LDL, followed by triglyceride hydrolysis. In addition to altering blood lipid levels, inflammation also impairs lipoprotein function. LDL becomes more susceptible to oxidation because HDL’s ability to prevent LDL oxidation diminishes. Furthermore, inflammation adversely affects several steps in the reverse cholesterol transport pathway. These lipids and lipoprotein abnormalities are consistently observed with greater severity in inflammatory diseases. Treatment that reduces inflammation typically restores the lipid profile towards normal. These changes in lipids and lipoproteins are integral to the innate immune response and likely play a crucial role in host protection during infection and inflammation [33]. TGs and DGs were observed as significant lipids in both studied groups, showing the ability to serve as indicators in both VAP severity and differentiation of *K. pneumoniae* from *S. aureus* bacteria.

In conclusion, there were significant differences in lipid metabolism in critically ill children with low and high VAP suspicion. The small sample size and the lack of an external validation cohort could be a limitation of the study; thus, the predictive performance of these lipids and their usefulness as biomarkers requires further verification. The study underscores the potential of lipidomics-based approaches to improve VAP diagnosis in PICU, necessitating further exploration of omics data integration with microbiological, molecular, and immune response studies. Such integrated investigations are crucial for elucidating the underlying biochemical pathways associated with VAP pathogenesis and diagnosis, offering insights into potential therapeutic targets and personalized treatment strategies.

## Figures and Tables

**Table 1:** Summarizes patients’ characteristics enrolled in the study. Patients’ characteristics adapted from [18].

	Total	High VAP Suspicion (mCPIS ≥ 6)	Low VAP Suspicion (mCPIS < 6)
	*n* = 20	*n* = 12	*n* = 8
**Age, months**	93 (6–184)	24.5 (6–141)	129 (28–184)
**male, *n* (%)**	13 (65)	9 (75)	5 (63)
**PRISM score**	12.1 (5.45)	13.1 (6.49)	11 (4.14)
**time to event, days**	9.37 (7.65)	8.73 (7.43)	8.73 (7.43)
**duration of mechanical ventilation, days**	26.3 (14.1)	24.4 (16.2)	29 (11)
**Hospitalization, days**	67.3 (47.4)	61.7 (46.0)	75.8 (51.2)
**mortality, *n* (%)**	2 (10)	2 (17)	0

**Table 2:** Characteristics of the constructed unsupervised and supervised models; UV scale was applied in all models.

Model	Type	N	R^2^X	R^2^Y	Q^2^	CV ANOVA
Samples_QC	PCA-X	131	0.580		0.262	
LOW vs HIGH VAP Suspicion	OPLS-DA	74	0.267	0.965	0.436	5.2 × 10^−6^
*K. pneumoniae* vs. *S. aureus*	OPLS-DA	30	0.286	0.987	0.528	4.8 × 10^−3^

**Table 3:** Lipids with statistical significance according to their VAP score (low and high suspicion).

Blood LOW HIGH Suspecion							
Lipids	*p* Value	*p* Value Adj. Waist	VIP	AUC	Log2FC	95% Lower CI	95% Upper CI
CE 18:3	6.99 × 10^−3^	3.32 × 10^−2^	1.07	0.65	−0.58	0.52	0.77
DG 36:3	2.34 × 10^−3^	1.62 × 10^−2^	1.41	0.73	−0.69	0.60	0.85
LPC 20:5	1.10 × 10^−2^	4.79 × 10^−2^	1.18	0.71	−0.62	0.57	0.81
PC 32:1	2.02 × 10^−3^	1.62 × 10^−2^	0.98	0.69	−0.52	0.56	0.80
PC 32:2	2.17 × 10^−5^	1.46 × 10^−3^	1.80	0.81	−0.69	0.70	0.91
PC 34:1	3.76 × 10^−3^	2.20 × 10^−2^	1.35	0.75	−0.27	0.63	0.86
PC 34:2	1.61 × 10^−3^	1.46 × 10^−2^	1.45	0.75	−0.33	0.62	0.85
PC 34:2	1.04 x 10^−2^	4.70 × 10^−2^	1.65	0.77	−0.33	0.66	0.87
PC 34:3	1.29 × 10^−2^	4.96 × 10^−2^	1.32	0.68	−0.96	0.57	0.80
PC 35:2	3.77 × 10^−4^	5.38 × 10^−3^	1.51	0.74	−0.49	0.62	0.84
PC 36:1	1.22 × 10^−2^	4.96 × 10^−2^	0.93	0.69	−0.31	0.56	0.79
PC 37:3	3.14 × 10^−3^	1.96 × 10^−2^	1.50	0.76	−0.37	0.65	0.87
PC 38:6	1.38 × 10^−3^	1.46 × 10^−2^	1.73	0.80	−0.87	0.69	0.90
SM 32:1	1.25 × 10^−2^	4.96 × 10^−2^	1.46	0.74	−0.60	0.61	0.84
SM 34:0	5.33 × 10^−3^	2.66 × 10^−2^	1.44	0.74	−0.43	0.63	0.85
SM 38:1	2.80 × 10^−4^	4.65 × 10^−3^	1.68	0.77	−0.56	0.66	0.86
SM 40:1	1.49 × 10^−3^	1.46 × 10^−2^	1.71	0.79	−0.62	0.68	0.89
SM 40:2	1.43 × 10^−3^	1.46 × 10^−2^	1.35	0.71	−0.41	0.59	0.82
SM 41:1	1.32 × 10^−4^	3.29 × 10^−3^	1.73	0.79	−0.59	0.68	0.89
SM 42:2	5.33 × 10^−3^	2.66 × 10^−2^	1.32	0.69	−0.44	0.57	0.81
TG 46:1	6.01 × 10^−5^	2.00 × 10^−3^	1.15	0.81	−1.09	0.69	0.90
TG 48:1	1.80 × 10^−4^	3.60 × 10^−3^	1.55	0.78	−0.85	0.66	0.87
TG 48:2	2.92 × 10^−5^	1.46 × 10^−3^	1.52	0.82	−1.39	0.72	0.91
TG 48:3	2.18 × 10^−3^	1.62 × 10^−2^	1.22	0.82	−2.08	0.72	0.91
TG 50:4	2.43 × 10^−3^	1.62 × 10^−2^	1.58	0.77	−1.32	0.65	0.87
TG 54:3	4.80 × 10^−3^	2.66 × 10^−2^	1.64	0.75	−0.88	0.64	0.86

**Table 4:** Lipids with statistical significance according to the bacteria strains *K. pneumoniae* and *S. aureus*.

Bacteria Strain						
Lipids	*p* Value	VIP	AUC	Log2FC	95% Lower CI	95% Upper CI
CE 20:4	3.45 × 10^−2^	1.1	0.68	−0.38	0.47	0.89
PC 33:2	1.27 × 10^−2^	1.7	0.75	−0.66	0.58	0.91
PC 34:3	1.47 × 10^−2^	1.2	0.71	−0.57	0.50	0.88
PC 36:4	1.76 × 10^−2^	1.4	0.73	−0.47	0.55	0.89
PC 36:4	2.46 × 10^−2^	1.2	0.73	−0.41	0.53	0.91
PC 37:3	6.23 × 10^−3^	1.9	0.76	−0.73	0.60	0.90
PC 38:4	2.97 × 10^−2^	1.4	0.72	−0.38	0.53	0.89
PC O-34:0	1.55 × 10^−2^	1.8	0.76	−0.40	0.59	0.91
PC O-38:4	3.29 × 10^−2^	1.7	0.72	−0.59	0.53	0.89
SM 34:1	1.04 × 10^−2^	1.7	0.67	5.76	0.48	0.87
SM 36:2	1.39 × 10^−2^	1.7	0.76	−0.35	0.57	0.91
SM 38:2	1.61 × 10^−2^	1.7	0.75	−0.32	0.56	0.91
SM 40:1	3.15 × 10^−2^	1.5	0.72	−0.44	0.53	0.88
TG 48:0	8.22 × 10^−3^	1.9	0.77	−0.27	0.61	0.92
TG 50:1	7.98 × 10^−3^	2.0	0.76	−0.60	0.58	0.91
TG 52:5	4.95 × 10^−2^	1.5	0.72	−0.76	0.51	0.88
TG 54:5	2.31 × 10^−3^	1.9	0.81	−0.76	0.64	0.95
TG 54:6	2.98 × 10^−3^	2.0	0.80	−0.36	0.63	0.93

## Data Availability

The original contributions presented in the study are included in the article/Supplementary Material, further inquiries can be directed to the corresponding author.

## Supplementary Materials

The following supporting information can be downloaded at: https://www.mdpi.com/article/10.3390/metabo14090466/s1, Table S1: Peak areas of annotated lipids.
